# Clinical characterization in a population of latin-American older adults using the frontotemporal dementia assessment scale: an observational study

**DOI:** 10.1186/s12877-026-08009-1

**Published:** 2026-08-07

**Authors:** Laura Sofia Aguilera-Ariño, Maria Alejandra Piñeros-Rendon, Maria Paula Patiño-Rattiva, Sofía López-Rodríguez, Suhamny Navas-Peñaranda, Mateo Avilés-Gomez, Paula Natalia Acosta, Juan Andres Ospina-Arias, Claudia Talero-Gutierrez

**Affiliations:** 1 School of Medicine and Health Sciences, Neuroscience Research Group (Neuros), Centro de Neurociencia - Neurovitae, Universidad del Rosario, Carrera 24 #63c -69, Bogotá, Colombia; 2 School of Medicine and Health Sciences, Undergraduate Neuroscience Research Group (Semineuros), Centro de Neurociencia - Neurovitae, Universidad del Rosario, Carrera 24 #63c -69, Bogotá, Colombia

**Keywords:** Aging, Cognitive Dysfunction, Frontotemporal Dementia, Cognition, Self-Care, Dementia

## Abstract

**Background:**

Aging is associated with gradual changes in cognitive, functional, and behavioral domains that may affect everyday functioning in older adults. In Latin America, these these functional and behavioral variations are frequently underrecognized in community settings due to limited access to standardized assessment tools. The Frontotemporal Dementia Rating Scale (FTD-FRS), validated in Spanish, allows allows the assessment of functional and behavioral changes related to daily activities and executive functioning beyond clinically diagnosed dementia. This study aimed to examine the presence and distribution of these changes in adults over 65 years participating in healthy aging programs in Bogotá, Colombia.

**Methods:**

This cross-sectional observational study was conducted among 80 adults aged 65 years or older who participated in community programs for healthy aging in Bogotá, Colombia. Individuals with a current diagnosis of cognitive impairment, dementia, or psychiatric disorders were excluded, and after providing written informed consent, the FTD-FRS, The FTD-FRS, consisting of 30 items that classify participants into six preservation categories, was administered. A descriptive analysis of the results was performed, while the associations between FTD-FRS preservation scores and sociodemographic variables were explored using JASP version 0.95.4.

**Results:**

Most participants showed variability in functional performance according to the FTD-FRS, with only 28.75% classified as normal/very mild, while the mild preservation category was the most prevalent in the population (41.25%). The domains “self-care and mobility” and “food preparation and consumption” were preserved in 93.1% and 89.5% of the population, respectively, while ‘behavior’ and ‘medication management’ showed the lowest preservation scores (75.5% each). Additionally, a trend toward lower FTD-FRS preservation scores was observed in participants with lower educational and socioeconomic levels, although without statistically significant differences.

**Conclusions:**

The application of the FTD-FRS in a population of community-dwelling older adults considered healthy and functionally independent demonstrated variability in functional performance across several domains of everyday functioning. Within the context of this exploratory study, the FTD-FRS proved to be a useful and accessible instrument for the descriptive characterization of functional performance. Future studies incorporating complementary cognitive, neuropsychological, and longitudinal assessments are needed to further evaluate its application in community settings.

## Introduction

Normal aging is a process in which gradual and heterogeneous changes occur across cognitive, functional, and behavioral domains, while basic activities of daily living are often preserved and more complex functions tend to decline progressively over time [1, 2]. Domains such as attention, temporal orientation, working memory, and planning are particularly vulnerable to age-related changes and are influenced by biological factors, multimorbidity, medication burden, and social determinants such as education and socioeconomic status [3, 4]. Distinguishing between these expected age-related trajectories and pathological changes remains a major challenge in geriatric and public health research, especially in community-based populations without formal clinical diagnoses.

Early frontotemporal alterations are often expressed through subtle changes in behavior, social cognition, motivation, and executive functioning rather than prominent memory deficits. These manifestations may include disinhibition, apathy, impaired judgment, reduced initiative, and difficulties in managing everyday tasks such as medication adherence, finances, and orientation in unfamiliar environments [5–7]. Neuroanatomically, these changes are associated with early dysfunction of the frontoinsular, anterior cingulate, and prefrontal-temporal networks, which can precede overt structural atrophy and the establishment of formal diagnostic thresholds [6, 8]. Longitudinal studies have shown that these early functional and behavioral changes may evolve gradually into clinically recognizable syndromes of frontotemporal dementia or other neurodegenerative disorders, underscoring the importance of detecting them in preclinical and community settings [7, 9].

Healthy aging programs provide a unique context for exploring early functional vulnerability in older adults who remain socially active and independent. These programs aim to promote physical activity, cognitive stimulation, and social engagement, all of which have been associated with greater cognitive reserve and delayed functional decline [1, 10, 11]. However, participation in such programs does not preclude the presence of subtle cognitive and behavioral alterations that may remain unnoticed in routine clinical care. Systematic functional characterization within these settings facilitates the identification of early risk patterns, informs targeted preventive strategies, and contributes to a broader understanding of aging-related vulnerability at the population level. From a translational perspective, this approach is of general interest because it bridges neuroscience, public health, and community-based intervention by highlighting how functional markers of frontotemporal systems can be explored outside specialized memory clinics and applied in aging populations across diverse sociocultural contexts.

Frontotemporal dementia (FTD) is classified as the third most common dementia in individuals over 65 years of age and is defined as a progressive neurodegenerative disorder affecting the frontal and anterior temporal lobes, with early involvement of behavior, personality, language, and executive functions [12]. Clinically, it encompasses several subtypes, including the behavioral variant (bvFTD), progressive non-fluent aphasia (nfvFTD), semantic variant (svFTD), and forms associated with motor neuron disease, such as corticobasal degeneration and progressive supranuclear palsy [13]. Neuroimaging studies have demonstrated characteristic patterns of cortical atrophy and hypoperfusion predominantly affecting frontal, temporal, and limbic regions, which are closely associated with functional and behavioral impairment [14–16]. Although FTD is traditionally studied in clinically diagnosed populations, the functional and behavioral domains assessed by instruments such as the Frontotemporal Dementia Rating Scale (FTD-FRS) have been examined across broader clinical and non-FTD groups, including individuals with Alzheimer’s disease, mild cognitive impairment, and healthy controls, as well as non-carrier family members in genetic FTD cohorts, supporting its utility for characterizing everyday functioning beyond formal FTD diagnoses [17, 18]. Growing evidence also suggests that alterations in attention, orientation, behavior, and executive functioning may emerge in older adults who are considered cognitively healthy and functionally independent, highlighting the need to better characterize functional vulnerability in community-dwelling populations [16, 19].

The assessment of functional and behavioral changes in everyday life is particularly relevant in Latin American settings, where dementia and non-Alzheimer neurodegenerative disorders are frequently underrecognized and access to specialized cognitive evaluation remains limited [20, 21]. Classical functional assessment instruments such as the Lawton & Brody Instrumental Activities of Daily Living (IADL) Scale have demonstrated good reliability and validity in Spanish-speaking older adults, supporting their use in geriatric research and community settings. This context helps situate the present functional characterization within the broader landscape of established functional assessment tools and their psychometric properties [22]. The Frontotemporal Dementia Rating Scale (FTD-FRS), originally developed as a staging instrument for FTD, has shown sensitivity to functional and behavioral disturbances across multiple domains of daily living and has been validated in Spanish-speaking populations [19, 20]. Beyond its role in grading disease severity, its structure allows for the systematic exploration of subtle functional changes related to frontotemporal cognitive systems in older adults without prior diagnoses. Therefore, this study aimed to determine the presence and distribution of functional and behavioral changes in adults over 65 years of age participating in healthy aging programs in Bogotá, Colombia, contributing to the understanding of patterns of functional performance and their potential sociodemographic correlates in a community-based Latin American context. 

## Materials and Methods

### Participants

A cross-sectional observational study was conducted among 80 adults aged 65 years or older and aimed to characterize patterns of functional and behavioral performance using the FTD-FRS in community-dwelling older adults. The study population consisted of older adults with no current diagnosis of cognitive impairment, dementia, or neuropsychiatric disorders who were able to provide written informed consent. Participants received standardized verbal and written information describing the study objectives, the voluntary nature of participation, confidentiality, anonymization procedures, and the approximate duration of the assessment. Those who agreed to participate provided written informed consent. These participants belonged to community programs organized by government agencies and private foundations in low- and middle-income areas. These healthy aging programs offered recreational and physical activities several days a week. All assessments were conducted in person at community centers where the healthy aging programs were held, and the surveys were administered by the research team. No electronic devices or screens were used by participants during data collection.

Convenience sampling was used. The data collection period extended from July to September 2025, and all data were entered into a secure Microsoft Excel database while preserving participant anonymity.

Recruitment was conducted through collaboration with coordinators of community-based healthy aging programs operated by local government agencies and private foundations in different neighborhoods of Bogotá. Potential participants were informed about the study during on-site informational sessions and through direct invitations issued by program staff. No financial or material compensation was offered for participation. A total of 102 individuals were approached, of whom 80 met the inclusion criteria and agreed to participate. Twenty-two individuals were excluded because they did not meet the age criterion, reported previous diagnoses of cognitive impairment, dementia, or psychiatric disorders, or declined to provide written informed consent.

Given the observational and survey-based design of the study, no blinding procedures were implemented. Data were collected by trained research assistants following a standardized protocol to minimize interviewer-related variability.

### Instruments

#### Sociodemographic survey

This survey collected information on the following variables: age, sex, place of birth, educational level, health insurance status, socioeconomic status, employment history, years of employment, years of unemployment, hobbies, and medical history.

#### Frontotemporal dementia rating scale (FTD-FRS)

The Spanish version of the FTD-FRS has been previously validated and was used in the present study to characterize patterns of functional and behavioral performance in everyday activities [5]. The instrument consists of 30 items distributed across seven functional domains (Table 1). Each item had four response options (“Always,” “Sometimes,” “Never,” and “Not applicable”) and was scored according to the frequency of the reported behavior, with the responses “Always” and “Sometimes” scored as 0 and “Never” scored as 1.

**Table 1:** Frontotemporal Dementia Rating Scale (FTD-FRS) Questionnaire

DOMAIN		ITEM
Behavior	1	Lacks interest in doing things – their own interests/leisure activities/new things
	2	Lacks normal affection, lacks interest in family members’ worries
	3	Is uncooperative when asked to do something; refuses help
	4	Becomes confused or muddled in unusual surroundings
	5	Is restless
	6	Acts impulsively without thinking, lacks judgement
	7	Forgets what day it is
Outing and Shopping	8	Has problems taking his/her usual transportation safely (car if has a driver license; bike or public transport if does not have a driver license)
	9	Has difficulties shopping on their own (e.g., to go to the local shops to get milk and bread if did not use to do the main shopping)
Household chores and telephone	10	Lacks interest or motivation to perform household chores that he/she used to perform in the past
	11	Has difficulties completing household chores adequately that he/she used to perform in the past (to the same level)
	12	Has difficulty finding and dialing a telephone number correctly
Finances	13	Lacks interest in his/her personal affairs such as finances
	14	Has problems organizing his/her finances and to pay bills (cheques, bankbook, bills)
	15	Has difficulties organizing his/her correspondence without help (writing skills)
	16	Has problems handling adequately cash in shops, petrol stations, etc. (give and check change)
Medications	17	Has problems taking his/her medications at the correct time (forgets or refuses to take them)
	18	Has difficulties taking his/her medications as prescribed (according to the right dosage)
Meal Preparation and Eating	19	Lacks previous interest or motivation to prepare a meal (or breakfast, sandwich) for himself/herself (rating based pre-morbid functioning; score same task for questions 19, 20 and 21)
	20	Has difficulties organizing the preparation of meals (or a snack if patient was not the main cook) (choosing ingredients; cookware; sequence of steps)
	21	Has problems preparing or cooking a meal (or snack if applicable) on their own (needs supervision/help in kitchen)
	22	Lacks initiative to eat (if not offered food, might spend the day without eating anything at all)
	23	Has difficulties choosing appropriate utensils and seasonings when eating
	24	Has problems eating meals at a normal pace and with appropriate manners
	25	Wants to eat the same foods repeatedly
	26	Prefers sweet foods more than before
Self-care and Mobility	27	Has problems choosing appropriate clothing (regarding the occasion, the weather or color combination)
	28	Is incontinent
	29	Cannot be left at home by himself/herself for a whole day (for safety reasons)
	30	Is restricted to the bed

The scores were summed and divided by the number of assessable items, excluding those marked as “Not applicable.” This yielded a total functional preservation score, which was subsequently converted into a logarithmic score (Table 2).

**Table 2:** Scores on the Frontotemporal Dementia Rating Scale (FTD-FRS)

Score %	Logit Score	Category	Score %	Logit Score	Category	Score %	Logit Score	Category
100	5.39	Normal / Very mild	66–63	0.88	Moderate	30–28	–1.27	Severe
99–97	4.12	Normal / Very mild	62–60	0.70	Moderate	27–24	–1.54	Severe
96–93	3.35	Mild	59–57	0.52	Moderate	23–21	–1.84	Severe
92–90	2.86	Mild	56–53	0.34	Moderate	20–17	–2.18	Severe
89–87	2.49	Mild	52–50	0.16	Moderate	16–14	–2.58	Severe
86–83	2.19	Mild	49–47	–0.02	Moderate	13–11	–3.09	Very Severe
82–80	1.92	Mild	46–43	–0.20	Moderate	10–7	–3.80	Very Severe
79–77	1.68	Moderate	42–41	–0.40	Moderate	6–4	–4.99	Very Severe
76–74	1.47	Moderate	40–37	–0.59	Severe	3–0	–6.66	Profound
73–71	1.26	Moderate	36–34	–0.80	Severe			
70–67	1.07	Moderate	33–31	–1.03	Severe			

#### Data quality control and bias reduction

To ensure data quality, all survey responses and FTD-FRS results were entered into a secure Microsoft Excel database, with continuous consistency checks performed by trained researchers. Selection bias and loss to follow-up were minimized through the strict application of the inclusion and exclusion criteria and by excluding participants who did not complete the assessment.

## Results

### Sociodemographic variables

**Fig. 1:**
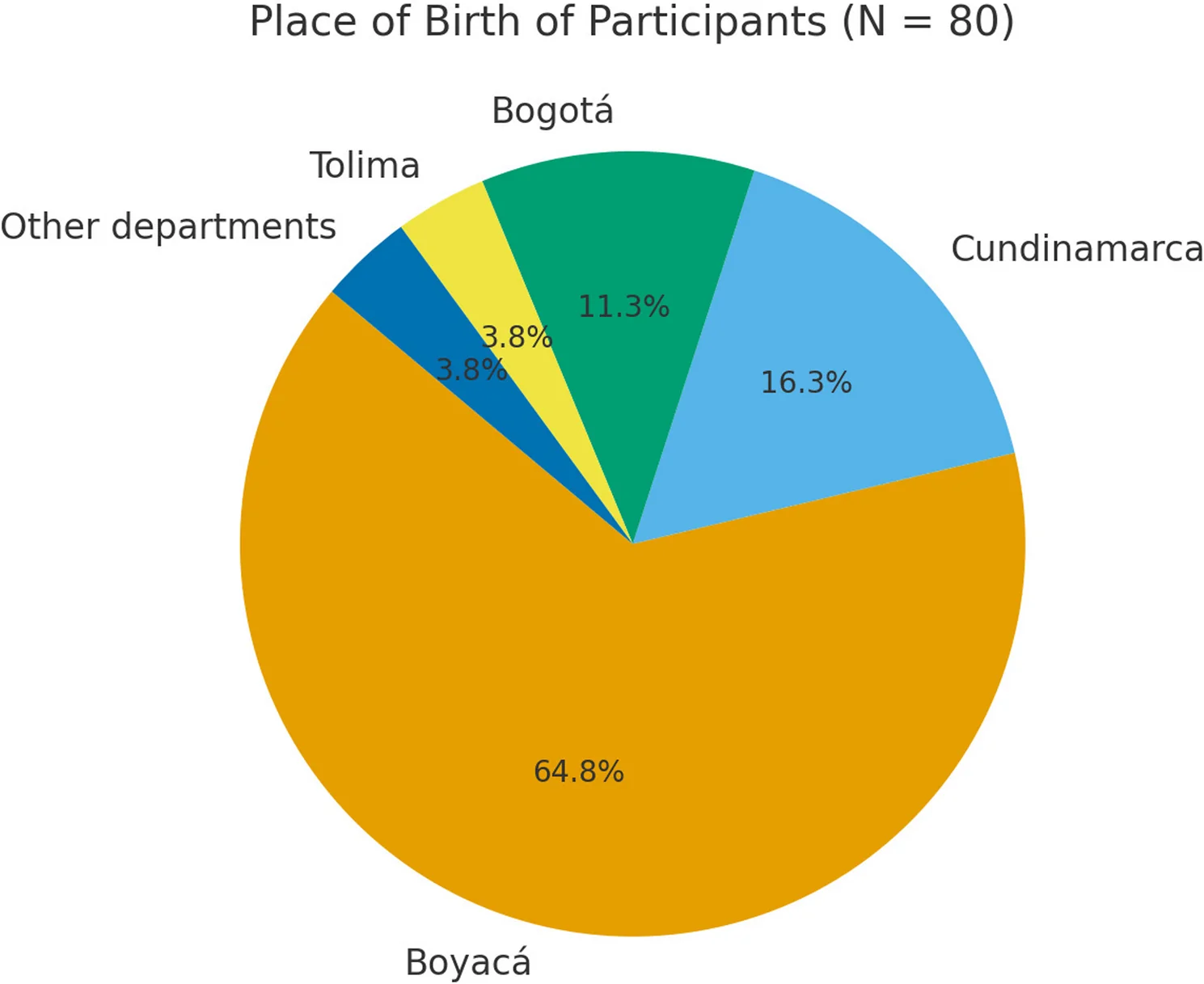
Distribution of participants by place of birth

total of 80 older adults were included in the study, with a mean age of 75.1 years (range, 60–93 years). Women comprised the majority of participants (73.8%; *n* = 59), while men accounted for 26.2% (*n* = 21). Regarding place of birth, the largest proportion of participants was born in Boyacá (52 participants; 64.8%), followed by Cundinamarca (13 participants; 16.3%), Bogotá (9 participants; 11.3%), Tolima (3 participants; 3.8%), and other departments (3 participants; 3.8%) (Fig. 1).

The study population was contacted through healthy aging programs in neighborhoods corresponding to different income levels. In terms of socioeconomic status, strata 1–2 that represents low income was the most frequent among the participants (58 people; 72.5%), followed by medium income, strata 3–4 (20 people; 25.0%), with little representation from the higher income, strata 4 and above (2 people; 2.5%). and in terms of health coverage 56.25% (45 people) belonged to the subsidized regime and 43–75% (35 people) to the contributory regime.

Socioeconomic distribution correlated with educational level. Just over half of the participants (44 people; 55.0%) attended primary school without completing it, while one-third managed to finish high school (26 people; 32.5%). A small group attained higher education (4 people; 5.0%), and 7.5% (6 people) never attended school.

Regarding previous occupations, the most common activity was domestic work (37 people; 46.25%), followed by general services (10 people; 12.5%) and agricultural work (6 people; 7.5%). The ratio of years of work activity to inactivity was close to 3:1, indicating that for each year of inactivity, approximately 2.9 years of work were performed.

Among hobbies, the most frequently mentioned activities were physical activity and sports (28 people; 35%), followed by household and craft activities (16 people; 20%), leisure activities (12 people; 15%), and other various hobbies (18 people; 22.5%). 7.5% of the subjects stated that they did not engage in any type of activity in their free time (6 people).

Additionally, the population’s medical history included: Diagnosis of high blood pressure (22.5%), diabetes mellitus (18.8%), dyslipidemia and hypothyroidism (8.75%), and others such as coronary heart disease, osteoarticular conditions (osteoarthritis, arthritis, and osteoporosis) and respiratory conditions (asthma, COPD, and obstructive sleep apnea syndrome), etc. (5%), and 15% of participants did not have any chronic conditions.

**Fig. 2:**
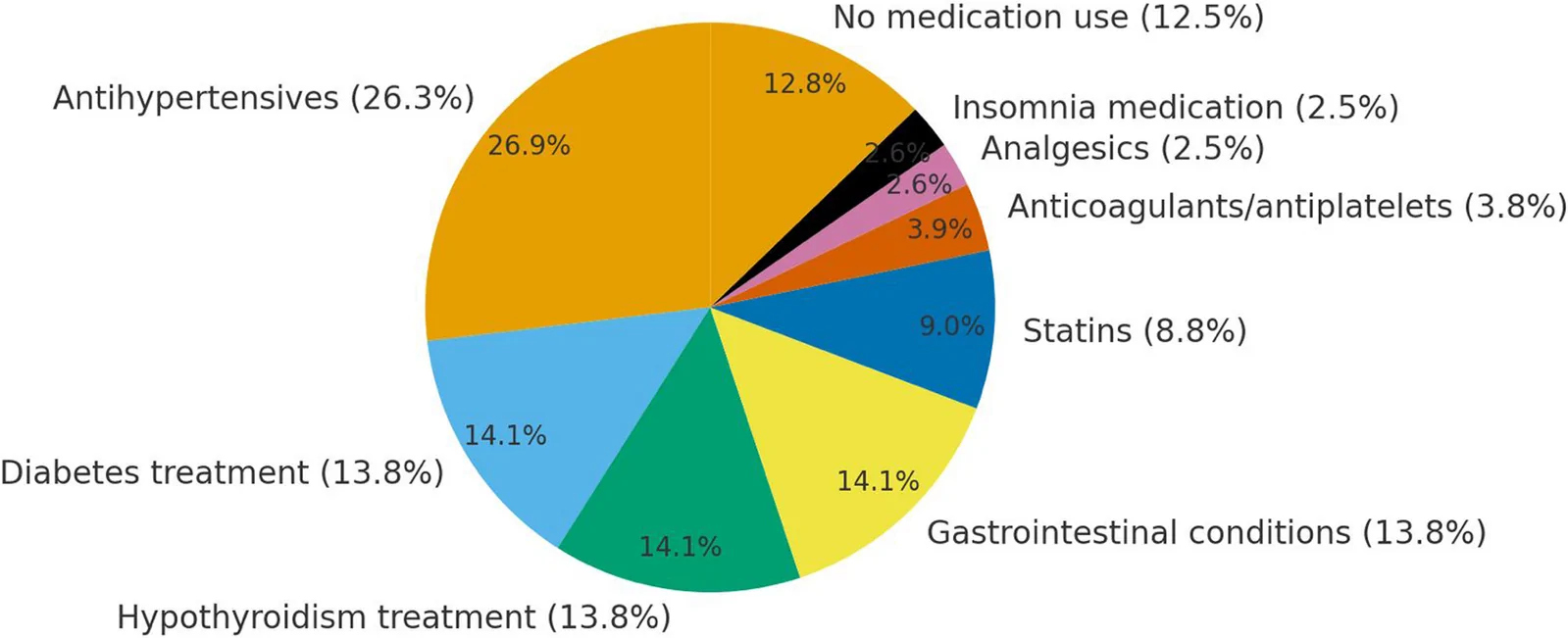
Medication use among participants

Consistent with this burden of disease, frequent use of medications was observed, notably antihypertensives in 26.3% (*n* = 21), 13.8% (*n* = 11) were being treated for diabetes, 13.8% (*n* = 11) for hypothyroidism, and 13.8% (*n* = 11) for gastrointestinal disorders. 12.5% of the sample (*n* = 10) reported not taking any medications. Less frequent use was found for statins (7 people; 8.8%), anticoagulants or antiplatelet agents (3 people; 3.8%), analgesics (2 people; 2.5%), and medications for insomnia (2 people; 2.5%). In this regard, most subjects (88.7%) used some type of chronic pharmacological treatment (Fig. 2).

### FTD-FRS

The Frontotemporal Dementia Rating Scale (FTD-FRS), adapted for the Spanish language, was applied to 80 adults over the age of 65 to characterize patterns of functional and behavioral performance. This allowed the participants to be classified into preservation score categories. The distribution of older adults was concentrated in the following preservation score groups: mild (96–80%), representing 41.25% (33 participants); normal/very mild (100–97%), representing 28.75% (23 participants); moderate (79–41%), representing 27.5% (22 participants); and severe (40–14%), representing 2.5% (2 participants). No participants were classified in the very severe or profound categories. Table 3 shows the percentages of functional preservation for each item and the total for each domain. These percentages were derived from the number of “Never” responses relative to the total number of applicable items, excluding responses marked as “Not applicable.”

**Table 3:** Results by Domain

DOMAIN	BEHAVIOR % (*N*)	OUTING AND SHOPPING % (*N*)	HOUSEHOLD CHORES AND TELEPHONE% (*N*)	FINANCES % (*N*)	MEDICATIONS % (*N*)	MEAL PREPARATION AND EATING % (*N*) (80)	SELF-CARE AND MOBILITY % (*N*)
ITEM	Domain total	Domain total	Domain total	Domain total	Domain total	Domain total	Domain total
ALL POPULATION 100% (80)	75.5%	83.2%	85.5%	87.8%	75.50%	89.50%	93.10%
NORMAL / VERY MILD 28.75% (23)	97.5%	97.7%	100.0%	98.9%	94.70%	100%	98.90%
MILD 41.25% (33)	81.00%	93.90%	91.70%	91.00%	85.40%	94.60%	93.20%
MODERATE 27.5% (22)	50.00%	53.50%	58.70%	72.90%	48.80%	75.00%	88.60%
SEVERE 2.5% (2)	14.30%	50.00%	0.00%	37.50%	100% (2)	37.50%	75.00%

In relation to educational level, it was observed that the proportion of participants with complete basic education or higher varied among the different preservation score groups. In the normal/very mild group, 29.17% (7 of 24 participants) had at least a high school education, compared with 9.68% (3 of 31) in the mild group, 13.04% (3 of 23) in the moderate group, and 50% (1 of 2) in the severe group.

n terms of socioeconomic status, most participants belonged to the lowest-income group, and proportional differences were also observed among the preservation score groups. Among those classified as normal/very mild, 58.33% (14 of 24 participants) belonged to the low-income group; in the mild group, 70.97% (22 of 31); in the moderate group, 78.26% (18 of 23); and in the severe group, 100% (2 of 2).

When analyzing the relationship between sociodemographic variables and FTD-FRS preservation score groups, no statistically significant differences were found for either educational level or socioeconomic stratification (*p* = 0.161, LR = 0.191; and *p* = 0.341, LR = 0.282, respectively). However, descriptively, there was a tendency toward a higher concentration of participants with lower educational levels and lower socioeconomic strata in the groups with lower FTD-FRS preservation scores, suggesting a descriptive pattern of social and educational vulnerability within the sample.

Regarding preservation scores, analysis of the entire sample showed that the domains with the highest proportion of participants with preserved function were “Personal Care and Mobility” (93.1%), followed by “Preparing Food and Eating” (89.5%). In contrast, the domains with the lowest preservation scores were “Behavior” and “Medication Management,” with preserved function observed in 75.5% of participants in each domain. At the individual item level, the lowest percentages of preserved function were observed for “Forgets what day it is” (68.8%), “Becomes confused and disoriented in unfamiliar environments” (70%), and “Problems taking medications at the right time” (70%) (Table 3).

Reviewing the participants classified in the “normal/very mild” preservation score group, it was observed that, across most of the evaluated domains, more than 95% of participants maintained preserved function. The only domain in which a slight decline was observed was “Medication Management,” where 94.7% of participants reported no difficulties, leaving 5.3% with some degree of difficulty in the items evaluated within this domain (Table 3).

Participants classified in the “mild” group retained preserved function in more than 90% of the evaluated domains. However, lower preservation scores were observed in the “Behavior” and “Medication Management” domains, where, on average, only 81% and 85.4% of participants, respectively, retained these functions. At the individual item level, the lowest percentages of preserved function were observed for “Forgets what day it is,” with 75.8% of participants responding “Never,” and “Lacks interest in doing things,” to which 84.8% responded “Never.”

A greater reduction in preservation scores was observed in the “moderate” group across all domains. The domains with the lowest proportion of preserved function were “Behavior,” with preserved function observed in approximately 50% of participants; “Medication Management,” with preserved function in 49%; and “Household Chores and Telephone,” with preserved function in 40% of participants. At the individual item level, 68% of participants reported forgetting what day it was, 59% reported becoming confused in unfamiliar environments, and 55% reported being restless and having difficulty taking their medications at the appropriate time (Table 3).

Finally, two participants were classified in the “severe” group. In these cases, marked reductions in preservation scores were observed across multiple functional domains. The domains with the lowest preservation scores were “Medication Management” (0%) and “Household Chores and Telephone” (0%). In addition, only 14% of the functions within the “Behavior” domain were preserved. The lowest preservation scores were observed in 11 of the 30 items, with complete loss of preserved function in at least two items across all domains except “Going Out and Shopping,” which showed 50% preservation in both items, and “Mobility and Self-Care,” which remained the best-preserved domain (75%) (Table 3).

## Discussion

Understanding functional and behavioral decline in aging is an important public health issue particularly in Latin America, a region with a high prevalence of cognitive impairment disorders such as FTD [23]. There is a particular need to better characterize functional and behavioral changes observed during aging, recognizing that these may reflect normal aging variability, sociocultural influences, or, in some cases, patterns that have also been described in neurodegenerative conditions. In this context, the application of instruments capable of characterizing variations in everyday functioning may contribute to a more comprehensive description of functional performance in community-dwelling older adults [24].

When applying the FTD-FRS to a community population of adults, a pattern of functional performance based on FTD-FRS scores was observed in the study population. Approximately 71.25% (57) of participants showed some degree of variation in functional performance according to the FTD-FRS, despite having no previous diagnosis of cognitive impairment, dementia, or psychiatric disorders, These findings should be interpreted cautiously, as the observed variability may reflect normal aging, individual differences, and sociocultural factors rather than clinically meaningful functional impairment. However, the distribution is mostly concentrated in the normal and mild levels of preservation scores. These findings illustrate the range of functional performance observed within this community-dwelling population and should be interpreted within the exploratory context of the study. Studies show that even older adults considered healthy may have fluctuations in executive functions such as attention, orientation, behavior and memory, which have been described both in normal aging and in neurodegenerative conditions, although they are not specific to either process [6, 7, 25, 26].

Although most participants showed variability in functional performance according to the FTD-FRS, 41% of the study population had preservation scores between 96% and 80%, corresponding to the mild preservation category. According to the scores obtained, the domains with the highest functional preservation were “self-care and mobility” (93.1%) and “food preparation” (89.5%). This finding is consistent with previous literature showing that, during normal aging, basic activities of daily living tend to remain relatively preserved, while motor and automated skills are generally maintained and abilities related to reasoning, information processing, and memory may show gradual age-related variability [1, 25]. In this context, lower FTD-FRS preservation scores may reflect differences in everyday functional performance and instrumental activities among community-dwelling older adults, although these findings should be interpreted descriptively and may also be influenced by normal aging variability and other individual or sociocultural factors. This interpretation is consistent with previous reports describing variability in everyday functioning during aging.

In contrast, one of the domains with the lowest FTD-FRS preservation scores was “behavior,” with 24.5% of participants showing differences in functional performance according to the FTD-FRS. The items with the lowest proportion of preserved responses were “Forgets what day it is” (68.8%) and “Becomes confused and disoriented in unfamiliar surroundings” (70%). These items assess aspects of temporal orientation and everyday executive functioning that have been described as susceptible to variability during aging and have also been reported in neurodegenerative conditions involving frontal and parietal networks [6, 26]. However, given the exploratory nature of this study and the absence of complementary neuropsychological or longitudinal assessments, these findings should not be interpreted as evidence of underlying neurodegenerative processes or disease progression [6, 26].

Similarly, lower FTD-FRS preservation scores were observed in the “medication” domain, involving 24.5% of participants. This finding deserves attention because medication management relies on cognitive processes such as memory, temporal judgment, and planning. Previous studies have reported that difficulties in medication management may occur in both normal aging and neurodegenerative conditions, although they are not specific to either process and should not be interpreted as evidence of underlying neurodegenerative disease in the present study [27–29].

These findings support the exploratory use of the FTD-FRS as a standardized instrument for describing patterns of everyday functional and behavioral performance in older adults. Within the context of this study, the scale provides complementary information on functional variability across domains of daily living and should be interpreted as a descriptive tool for functional characterization rather than as evidence of disease-specific changes [24, 25].

In relation to sociodemographic factors, although no significant relationships were found between the preservation score groups and variables such as educational level and socioeconomic status, the descriptive results suggest a trend toward a higher concentration of participants with lower educational levels and socioeconomic status in the lower FTD-FRS preservation scores. This descriptive pattern may reflect the influence of social and educational factors on everyday functional performance within the study population, although these observations should be interpreted cautiously given the exploratory nature of the study.

The literature documented that high levels of education and socioeconomic status act as protective factors against cognitive decline, promoting greater cognitive reserve and better coping strategies for brain aging [1, 3, 4]. In contexts of lower educational attainment, greater social vulnerability, reduced exposure to stimuli that develop new intellectual abilities and structural inequalities can accelerate cognitive decline [1, 3]. Taken together, these findings suggest that the descriptive patterns observed in the present sample may reflect differences in everyday functional performance influenced by social and educational determinants, rather than evidence of underlying neurodegenerative processes.

Another relevant point is the prevalence of chronic comorbidities in this population, mainly hypertension, diabetes and dyslipidemia, and the use of chronic medication in 90% of them. It has been established that multimorbidity and polypharmacy increase the risk of cognitive decline through vascular, inflammatory, and metabolic mechanisms (10, 28–29). Alterations in cerebral perfusion, synaptic dysfunction and some pharmacological side effects can compromise cognitive tasks such as attention and memory, which may contribute to variability in everyday functional performance among older adults [1, 29]. These results highlight the importance of considering medical and social factors when assessing the functional performance of older adults .

Despite the vulnerabilities, a large majority of participants had some form of physical, social, or cognitive hobby, and most retained their basic autonomy and independence in essential activities. This finding is consistent with evidence showing that interventions such as cognitive stimulation, physical activity, and social habits are protective factors for maintaining daily skills [10, 26]. Regular participation in stimulating activities has been associated with the activation of neural networks related to attention, planning, and working memory, contributing to the maintenance of functional autonomy, which is one of the fundamental pillars of healthy aging and may also contribute to overall well-being [11].

In the Latin American context, the use of the FTD-FRS is particularly relevant. Studies have shown that frontotemporal dementia and non-Alzheimer dementias are frequently underdiagnosed due to the lack of specific instruments and limited training in the recognition of frontal syndromes [23, 24]. The present study supports the usefulness of applying culturally adapted tools in the Latin American population, where subtle variations in everyday functional and behavioral performance may be confused with changes attributed to normal aging. Consistent with the exploratory nature of this study, the analyses were intentionally limited to descriptive statistics, and no inferential testing or multivariable adjustment for potential confounding factors was performed. Within this context, the FTD-FRS provides an accessible and low-cost approach for the descriptive characterization of executive and behavioral domains, supporting greater awareness of functional variability among older adults and informing future studies using complementary assessment strategies.

## Conclusions

The application of the FTD-FRS scale in a sample of older Colombian adults with no previous diagnosis of neurological or psychiatric disease showed that a substantial proportion of participants presented different patterns of functional performance according to the FTD-FRS, despite being considered clinically healthy and independent. In the present study, the FTD-FRS was not used as a diagnostic or screening instrument, but rather as a standardized framework to characterize patterns of everyday functional and behavioral performance in a community-dwelling older adult population. This exploratory approach is intended to generate preliminary data to inform future studies that will incorporate complementary cognitive, neuropsychological, and behavioral measures to formally assess convergent and discriminant validity. Within this exploratory context, the results support the use of the FTD-FRS as a useful and accessible tool for the descriptive characterization of functional performance in older adults, rather than for screening or predicting neurodegenerative conditions. Likewise, the findings on educational and sociodemographic levels highlight the need to consider other social determinants as factors that influence functional performance.

### Limitations of the study

This study has limitations that should be considered when interpreting the findings. A convenience sampling strategy was used, limiting the ability to generalize results to broader populations of older adults. The cross-sectional design precludes causal inference or temporal interpretation of observed patterns. The absence of complementary cognitive and emotional assessments, such as standardized cognitive screening tools or mood questionnaires, limits the capacity to differentiate between normal age-related variation and potential underlying cognitive or affective conditions.The general aspect of the findings is constrained by the specific community context and sociodemographic characteristics of the sample. Finally, considerations related to instrument validity is warranted, as the application of the FTD-FRS outside clinically diagnosed populations may not fully capture clinical constructs; future studies should incorporate multimodal and multi-instrument approaches to allow for cross-validation and a more comprehensive assessment of early functional decline 

.

## Data Availability

The datasets used and or analyzed during the current study are available from the corresponding author on request.
